# A unique physeal injury of the distal phalanx

**DOI:** 10.3109/23320885.2014.999780

**Published:** 2015-01-06

**Authors:** Onur Berber, Bijayendra Singh

**Affiliations:** ^a^ Medway Maritime Hospital, Gillingham, Kent, UK

**Keywords:** Salter–Harris, phalanx, fracture, rare

## Abstract

An unusual Salter–Harris Type 1 fracture variant of the distal phalanx of the index finger is described. The epiphysis was dislocated, sitting dorsally over the middle phalanx head with the articular surface facing dorsal. Reduction could only be achieved through an open procedure. The reduction was stable without supplemental fixation.

## Introduction

Physeal injuries are commonly seen in children and according to Fischer and McElfresh (1994), the Salter–Harris Type 2 are the most frequent [1]. In contrast Salter–Harris Type 1 injuries are less frequently seen. Reports in the literature commonly describe cases with delayed presentation because the injuries were missed in the acute setting. The importance of prompt diagnosis and treatment of physeal injuries cannot be overstated enough. The aim of this report is to highlight such a case.

## Case report

**Figure 1.**
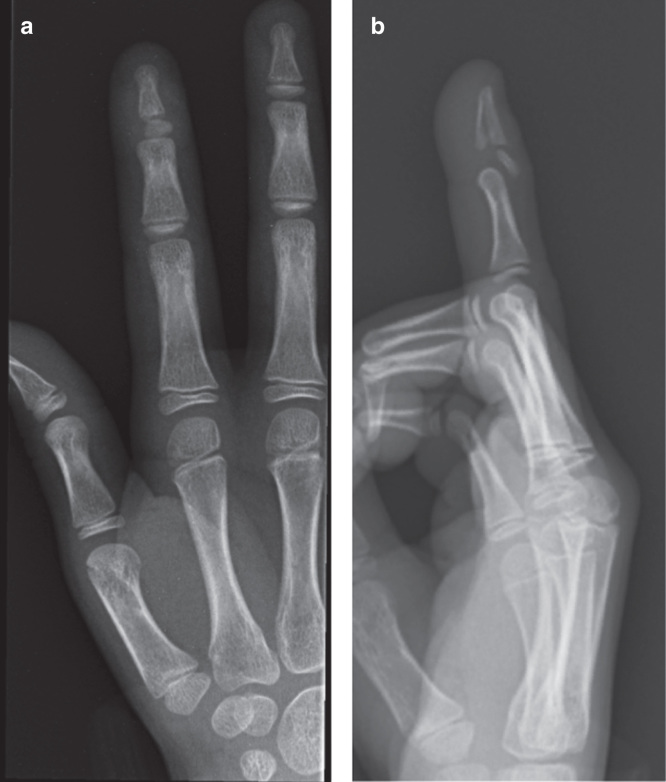
**Anteroposterior and lateral radiographs demonstrating a Salter–Harris Type 1 fracture to the right index finger distal phalanx.**

An 8-year-old girl presented with an injury to the index finger of her dominant hand. Injury occurred while playing netball and presentation was on the day of injury. The distal interphalangeal joint was swollen and tender with a loss of active movement. Radiographs showed a physeal injury of the distal phalanx with an associated fracture split extending to the tip (Figure 1*a* and *b*). The epiphysis was dislocated having slipped dorsally.

**Figure 2.**
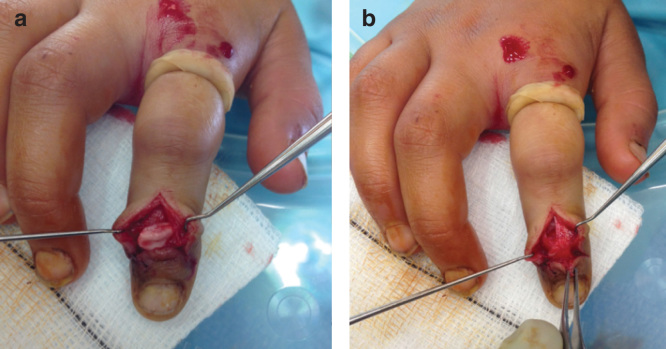
**Intra-operative images demonstrating the dorsally displaced epiphysis (*a*) and appearances once reduced (*b*).**

**Figure 3.**
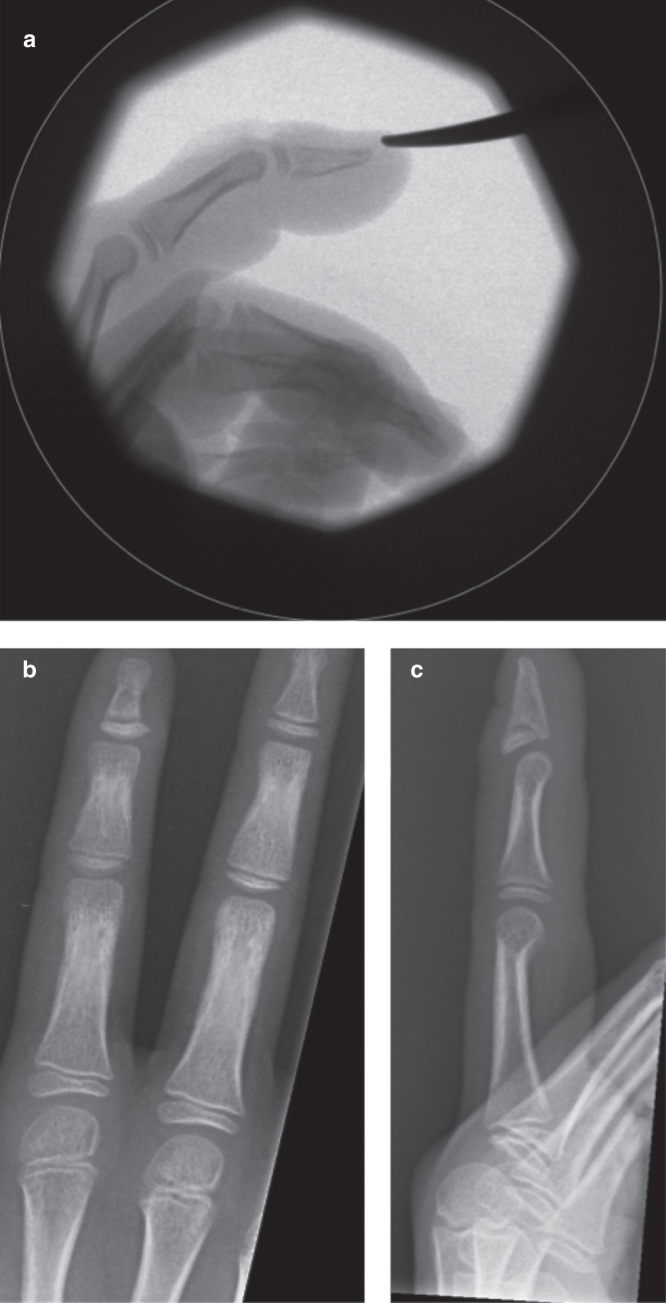
**Intra-operative radiograph demonstrating reduced epiphysis (*a*). Post-operative radiograph at 6 weeks indicating subtle displacement of the epiphysis (*b and c*).**

**Figure 4.**
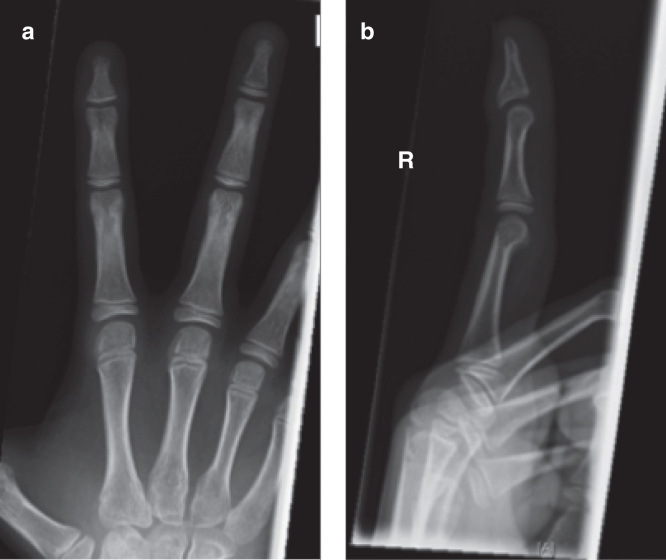
**Radiographs at 2 years demonstrating good coronal alignment on the anteroposterior view (*a*). However, premature fusion of the physis has occurred with a degree of malunion on the lateral view (*b*).**

The patient was taken to theater soon after presentation and an attempt was made at closed reduction. Manipulation maneuvers failed and instead caused further rotation and displacement of the epiphysis. The joint was therefore opened dorsally through a cruciate incision to enable reduction. The epiphyseal fragment was found to be lying between the head of the middle phalanx and the extensor hood central slip. The articular surface was rotated nearly 180° and facing the fingertip (Figure 2*a*). Some of the central extensor slip remained attached to the distal phalanx. Reduction occurred without significant force and was stable in flexion and extension. Therefore, no fixation was required (Figure 3*a*). Radiographs taken at 6 weeks demonstrate some extension type displacement of the epiphysis (Figure 3*b* and *c*). At 2 years, the radiographs illustrate premature fusion of the physis with a degree of malunion (Figure 4*a* and *b*). Function at this stage was good and range of movement −5 to 50 degrees.

## Discussion

**Figure 5.**
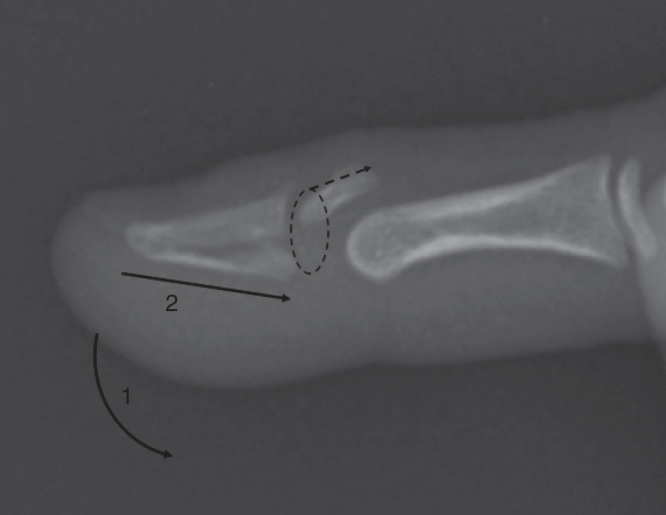
**Illustration representing proposed mechanism of displacement. Axial loading (lines 1 and 2) in extension could lead could lead to dorsal dislocation of the epiphysis (dotted lines).**

It was assumed that the mechanism of fracture was caused by axially loading the finger from the tip with the distal interphalangeal joint in hyperextension. Loading the finger in this position would enable the head of the middle phalanx to displace the epiphysis and fracture the distal phalanx (Figure 5). The position of the dislocated epiphysis lying between the head of the middle phalanx and the extensor mechanism meant that closed reduction was not possible. Longitudinal traction led to tightening down of soft tissue onto the fragment. Intra-operative findings of the epiphysis rotated 180° suggest that reduction maneuvers further displaced the fragment from its position seen in the pre-operative radiographs (Figure 1*a* and *b*). It is therefore recommended that only a gentle reduction be first attempted, and if this fails, early open reduction should be performed. This would minimize further injury to the epiphysis and its soft tissue attachments. There have been reports of similar injuries in published literature but none in this specific pattern [2, 3, 4, 5, 6, 7]. The injuries were delayed presentations in all but one case and an open procedure was typically required to reduce the displaced epiphysis [3]. In one case, an external fixator is used to distract the joint for a period prior to a formal open reduction [7]. These injuries appear to be commonly missed, especially if the injury happens in the very young prior to appearance of the epiphyseal ossific nucleus [6]. Therefore, a high index of suspicion should be exercised when managing childhood these physeal injuries.
